# Trauma care systems in Saudi Arabia: an agenda for action

**DOI:** 10.4103/0256-4947.59374

**Published:** 2010

**Authors:** Mohammed Y. Al-Naami, Maria A. Arafah, Fatimah S. Al-Ibrahim

**Affiliations:** a From the Department of Surgery, King Khaled University Hospital, Riyadh, Saudi Arabia; b From the Department of Pathology, King Saud University, Riyadh, Saudi Arabia; c From the Department of Surgery, King Saud University, Riyadh, Saudi Arabia

## Abstract

Saudi Arabia is undergoing a rapid population growth that along with improved socioeconomics has led many individuals to own a car or even a number of cars per family, resulting in a greater number of vehicles on the roads. The reduced focus on good public transportation systems and the dependence on cars for transportation have created a diversity of drivers who are unfamiliar with the local driving rules and lack the basic skills for safe driving. This is in addition to some young drivers who frequently violate traffic laws and tend to speed most of the time. This unplanned expansion in road traffic has resulted in more car accidents, injuries, disabilities, and deaths. Accompanying that is an increased socioeconomic burden, depletion of human resources, emotional and psychological stress on families, and a strain on healthcare facilities. If this continues without prompt intervention, it will lead to increased insurance premiums and may become unmanageable. To minimize this impact, a national or regional multidisciplinary trauma system has to be developed and implemented. A trauma system is a preplanned, comprehensive, and coordinated regional injury response network that includes all facilities with the capability to care for the injured. Essential components of the system include trauma prevention, prehospital care, hospital care, rehabilitation, system administration, trauma care education and training, trauma care evaluation and quality improvement, along with the participation of society. Research has documented a significant decrease in morbidity and mortality from trauma after the implementation of such systems, depending on their efficiency. The purpose of this review is to discuss the problem of road traffic accidents in this country and address the trauma care system as an effective solution.

Urban development in Saudi Arabia resulting from the oil boom and the sharp rise in living standards has brought about dramatic changes to the road network and an increase in the number of cars. This rather abrupt change has contributed to an increase in the number of road traffic accidents (RTAs) and a multiple-fold rise in the number of injuries and deaths over the last few decades.^1^,^2^ National efforts are not up to expectations for coping with these problems. These efforts have been mostly directed toward limited changes in traffic laws and improving roads, not addressing the root causes. The legal regulatory framework is not well-enforced and minimal attention is paid to changing driver behavior, which is considered the most important and most difficult factor to control.^3^,^4^ Many programs and campaigns have failed to re-educate drivers, probably because they were targeting the wrong audience. The way people live is the way they drive.^5^ To bring about positive behavioral changes, attention should be directed towards young people before they are actually allowed to drive.^6^,^7^ The aim of this review is to discuss the magnitude of the problem of road traffic accidents (RTAs) in Saudi Arabia and suggest the establishment of a trauma care system as a solution to improve trauma outcomes.^8^–^10^

## Socioeconomic and medical effects of RTAs

Road traffic accidents (RTAs) or motor vehicle accidents (MVAs), when fatal or otherwise, are a socioeconomic burden on the victims and their families and on the hospitals and rehabilitation centers. Trauma deaths tend to deplete the pool of human resources as the majority of the victims are working males.^11^ It is difficult to estimate the economic cost of RTAs as it entails medical care, rehabilitation services, and the loss of productivity as a result of absenteeism or disability.^12^ In the city of Jeddah alone, the cost of road fatalities in 1987 was estimated to be 648.7 million Saudi Riyals (USD $172.5 million).^13^ A conservative estimate of the annual cost of RTAs to the country is 21 billion Saudi Riyals (USD $5.6 billion), an estimated loss of 2.2% to 9% of the national income.^1^ The loss of an earning member of a family leads to a change in household dynamics, causing financial and emotional losses.^1^,^14^ Some of those who lose their ability to earn a livelihood on account of a disability are burdened with their own healthcare, and a number resort to selling most of their assets and may even end up in bankruptcy.^14^ About 3000 people worldwide die every day from road traffic injuries.^14^ Low- and middle-income countries account for about 85% of the deaths and for 90% of the annual disability-adjusted life years (DALYs) lost because of road traffic injury.^14^ Trauma is the leading cause of mortality and morbidity in young age groups in Saudi Arabia^1^ with RTAs accounting for 80% to 85% of these traumas.^15^ In 1986, the death rate from RTAs was 26.5 per 100 000 of the resident population.^13^ It has been estimated that in this country, one person is killed and four are injured every hour.^1^ The traffic data for 2005 have shown that the number of RTAs in Saudi Arabia was 283 684 with 5883 deaths; half of them were below 30 years of age, and around 60% were Saudis.^16^ RTAs are most common in the Makkah region followed by the Eastern province.^16^ The highest incidence of RTAs was found on four major roads: Dammam-Riyadh road, Dammam-Jubail road, Riyadh-Taif road, and Riyadh-Qassim road.^17^ At any given time, about one-fifth of Ministry of Health hospital beds are occupied by road traffic victims.^1^ Many of these patients have residual disabilities requiring long-term rehabilitation care, which is suboptimal in most regions. Approximately 74% of all cases of hemiplegia, paraplegia, and quadriplegia in Saudi Arabia are due to road traffic accidents.^1^ Head and facial injuries account for 30% of all injuries and cause 26% of deaths.^1^ Musculoskeletal and central nervous system injuries require the highest care and contribute to prolonged hospital stay.^18^ Blunt abdominal trauma is a common injury, especially in children, with the liver and spleen being the most frequently injured solid organs in 91% of cases.^19^ Blunt trauma from RTAs is the most frequent cause of chest injuries in children, with the lung being the most commonly injured intrathoracic structure.^20^ The relative compressibility of the child's thorax accounts for the severe intrathoracic injuries without rib fracture following a crushing force.^20^ Hemorrhage from trauma is a major life-threatening condition in children. When hemorrhage coexists with thoracic injury, mortality increases to as high as 25% from 5% for hemorrhage alone. In the presence of head injuries, the mortality may reach 100%.^21^–^23^

## Reasons behind soaring numbers of road traffic accidents

The increase in number of vehicles and expansion of road networks within and between cities and towns have contributed to the increasing number of RTAs in Saudi Arabia. Generally, traffic accidents are attributed to one of three major factors: the human factor, the vehicle, and the road/environment.

### Human-related causes

Driver errors account for about 80% of all RTAs in Saudi Arabia.^16^ Overspeeding is responsible for 65% of all traffic accidents (3.5 times the incidence in the USA).^1^ Violation of traffic signals at urban intersections is responsible for about 50% of accidents (4.5 times more common than in the USA).^1^,^3^ Other errors include passing another vehicle or overtaking from the wrong side (eight times more common than in the USA), driving without a license, incorrect parking, and incorrect U-turns.^1^,^16^ Another often neglected cause is the poor maintenance of vehicles and tires. Although 90% of the drivers have claimed that they check their tires at least once a month, it was found that most of them did it incorrectly.^17^ Furthermore, only 23% of the drivers knew the correct tire pressure recommended by the manufacturer for their vehicles.^17^ Use of cellphones while driving is a deadly mixture, especially distracts the driver's attention from driving.^24^ Another issue of major significance is noncompliance with traffic laws, especially the wearing of seat belts. Although the “wearing seat belt” law was made compulsory in Saudi Arabia on December 5, 2000, only 4% of lower socioeconomic-class passengers and about 40% of middle-class passengers were found to abide by this law.^2^ Most traffic fatalities are due to severe head injuries that are mostly related to the non-use of seat belts.^25^ About 40% of RTAs are attributed to non-Saudis.^16^ Thus, the increasing number of expatriates from different countries who are unfamiliar with local driving conditions and requirements is also an important factor in the increasing number of RTAs in the country.^1^ Stress and exhaustion due to long working hours, moving to larger cities, poor education, young age, and aggressive personality traits were also found to be associated with a greater number of accidents.^6^ Other general factors related to people include poor child restraint and unsupervised children playing in the streets, resulting in injuries as pedestrians.^26^ Among other important contributing factors are unfamiliarity with vehicles, thrill-seeking, and overconfidence.^14^ In Saudi Arabia, there is an obvious lack of a good attitude and common courtesy among some drivers who believe that they always have the right of way and every one has to yield to them. For example, instead of lining up at a traffic light, they move in front of other cars and position themselves close as possible to the traffic light. This unacceptable practice is more common among the affluent and young drivers.

### Vehicle, road and environment-related causes

Vehicles and road layouts contribute to accidents and account for 20% of RTAs in Saudi Arabia.^16^ People in small and light vehicles are more likely to have serious injuries and fatalities.^17^ Improving vehicle manufacturing should result in less fatal road traffic injuries.^14^ Tire blowouts and poor roads are very “hot” safety issues in Saudi Arabia, particularly in rural areas. The total number of tire-related accidents occurs at a rate of one accident per 11 km of rural roads according to a 2001 report on traffic accidents.^17^ Another problem is that some four-lane roads suddenly become three-lane roads after an intersection or lane-marking deviations. Drivers have a hard time adjusting to this, especially if the light at the intersection is green and there is no time to adjust. Traffic signals are lacking in some smaller roads, and right-of-way is unclear. Ongoing highway construction at entrances and exits from and into service roads is another problem. Collisions are common when highway entrances and exits are in close proximity (half a kilometer) and there is little room to maneuver.

At peak times, vehicles are not allowed to enter highways at some entrances, resulting in more congestion in service roads, which defeats the purpose of having highways, which are supposed to reduce congestion. Among solutions to minimize congestion at peak times are the use of one or two lanes from the opposite side of the free highway. Environmental factors such as rain, fog, and dust have minimal effects on RTAs in Saudi Arabia.^4^ However, extreme heat is responsible for 39% of all accidents due to tire blowouts.^27^ Heat also contributes to driver stress levels, leading to reduced mental capacity.^28^

## Ambulance services

In Riyadh, there are 7 ambulance stations with only 14 ambulances and fewer than 30 emergency medical technicians (EMTs) to cover an area of 4 million people.^29^ This is not enough to serve such a highly populated urban area. In South Carolina, for example, there are 478 ambulances with 2542 active state-certified EMTs to cover an area of 3.1 million people.^29^ Most of the emergencies in Saudi Arabia are transported either by volunteers or police vehicles, not by the Saudi Red Crescent (SRC) ambulances. There is a lack of reliance on the SRC that may be due to a lack of confidence in the service or a lack of awareness of its role. According to an SRC survey (Magazine of SRC Society, 1998), only 3% of those interviewed had recognized that the emergency phone number for SRC is 997, 70% had answered that it was 911, which they knew from the famous 911 US television program.^29^ Although an air medical evacuation system is available between cities in Saudi Arabia, this service is limited to some seriously ill patients but is not enough to cover major injuries. In addition, getting the service is a complex process that is also relatively time-consuming and hence, a structured helicopter emergency rescue service is under study. This service is more important for the rapid evacuation of seriously injured victims to appropriate trauma facilities. In Germany, for example, helicopter evacuation of seriously injured patients to a trauma care facility is usually carried out within 15 minutes.^30^

## The need for improved trauma care

The need for improved trauma care is obvious. Statistically, trauma is ranked as the number one killer in Saudi Arabia;^1^ RTAs accounting for 80% to 85% of these traumas.^15^ In 1986, death due to RTAs was found to be 26.5 per 10 0000 of the resident population.^13^ In 2005, the number of RTAs in Saudi Arabia were 283 684 and resulted in 5883 deaths.^16^ Countrywide prehospital care is suboptimal with the exception of some centers in big cities and on major roads. Rehabilitation services are also deficient, except for a few centers. Prevention, communication, transportation, hospital care, training, and public education need further emphasis.

## What is the solution?

After discussing the magnitude of the problem of RTAs in Saudi Arabia, it is obvious that the care of injured people in this country is not up to the desired standards. There have been calls for urgent and aggressive interventions to save lives and resources. RTAs involve many factors that need to be addressed collectively by all facilities that are concerned with this escalating problem. Research^8^,^9^,^10^,^31^,^32^ and reports^33^,^34^ in some countries have shown that implementation of trauma systems can significantly decrease mortality and morbidity and even the cost^35^ due to trauma based on the system's efficacy. For these reasons, the development and implementation of trauma care systems in Saudi Arabia have been deemed necessary in order to improve trauma care and outcome. Sectors and institutions concerned with trauma and related problems should work together to study and discuss the feasibility of developing and implementing such a system.

## What is a trauma system?

A trauma system is a planned, comprehensive, and coordinated region- or countrywide injury response network that includes all facilities and sectors with the capability to care for the injured, and is integrated with the local public health care system. This system involves components related to trauma prevention, prehospital care, hospital care, rehabilitation of the disabled, disaster medical planning, systems administration, trauma care education and training, trauma care evaluation and total quality improvement, and includes the participation of society. A country or a region should set their own goals according to the resources and facilities available. Ideally, a successful trauma system should involve the following goals:^34^

- To decrease the incidence and severity of trauma
- To ensure optimal, equitable, and accessible care for all persons sustaining trauma
- To prevent unnecessary deaths and disabilities from trauma
- To contain costs while enhancing efficiency
- To implement quality and performance improvements in trauma care throughout the system
- To ensure that certain designated facilities have appropriate resources to meet the needs of the injured

**Figure 1:**
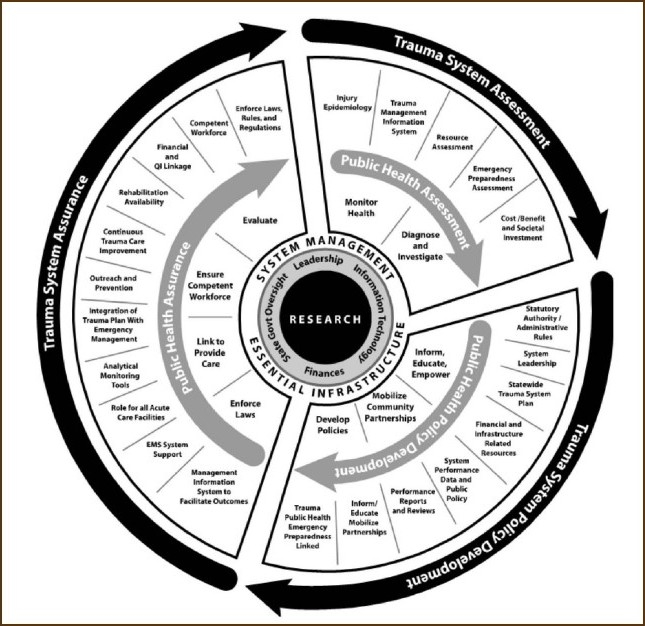
Core functions and essential services of the trauma system. *From Health Resources and Services Administration. *Model Trauma System Planning and Evaluation*. Rockville, MD: Health Resources and Services Administration; 2006:18.^34^

These goals can be achieved collectively at once or gradually in steps depending on the local resources. The core functions and essential services of the trauma system are summarized in Figure 1.

## Essential components of a trauma system

The first component of a trauma system is the identification of risk factors leading to injury and it needs assessment through data collection and research. After establishing the need to improve trauma care, public information and education are initiated through prevention programs and the establishment of a trauma advisory committee. A regional trauma advisory committee representing all sectors involved in the care of the injured will be responsible for trauma system leadership, legislation, and planning. Prehospital care involves communication, triage and transport to a trauma care facility, medical direction, and treatment protocols. Definitive care refers to patient management in designated trauma care facilities, interfacility transfer, and rehabilitation of the injured. Human resources are provided through workforce resources and educational preparation. System evaluation and monitoring are achieved by ongoing research through a trauma registry and are based on quality improvement data.

## The trauma advisory committee (lead agency)

A trauma system is best managed through a national or regional trauma advisory committee (TAC). Trauma-concerned community sectors should be approached to introduce them to the rationale behind planning and implementation of a trauma care system and to ensure their willingness to cooperate and participate in developing it. As trauma is an ethical and legal responsibility of the medical profession,^36^ the TAC should be led by a full-time or part-time trauma medical manager and medical representatives from universities, medical schools and university hospitals, the Ministry of Health, Saudi Red Crescent, the military, Security Forces, National Guard, and private hospitals involved in trauma management. Representatives of the Police and Traffic Regulation Authority, Civil Defense, Ministry of Transportation, and Ministry of Labor and Social Affairs are also important. The TAC is the lead agency that sets legislation, enforces policies and laws, plans and develops the system, assures integration and collaboration among sectors, builds coalitions and partnerships that can assist in ensuring injury prevention, and monitors system efficacy. Other important leaders in the system include prehospital trauma care directors, trauma healthcare facility directors, rehabilitation care directors, and others. Those leaders work with the lead agency to educate and train people, ensure the implementation of the system, and assist in trauma system evaluation and research.^34^ The lead agency should be supported by the government, with adequate resources that include an office for the trauma system program manager, secretarial assistance, a meeting room, data entry and analysis personnel, monitoring and law enforcement personnel, epidemiologists and researchers, and others as needed.^37^

## Legislation

Legislation consist of rules and regulations set by the lead agency.^34^ These may include traffic laws, rules and regulations at all levels and components of the trauma care system, and methods for their implementation. The TAC may review current legislation, modify or add new legislation, and consider their feasibility as necessary to suit national and/or regional circumstances. These rules and regulations should be applied with equal enforcement so that no one is above the law, and with continuous monitoring and periodical evaluation. Legislation should also enforce the integration and collaboration of services concerned with trauma so that a comprehensive and coordinated system can be successfully implemented with some efficiency and durability.

## System planning

The planning starts with the commitment of the lead agency members and corresponding sectors involved in the care of the injured.^34^ Members should have the same mindset on common philosophy and future vision for the system, share the same goals and objectives, coordinate to bridge differences in opinions, and attempt to reach a consensus for all issues.^37^ Lead agency members should have a diversity of skills and experience in management, education, and clinical practice. A plan should be produced by the lead agency and other concerned agencies based on the assessment of its needs, expected challenges, known gaps and barriers, and ongoing updates. The plan should be integrated and inclusive of prevention programs, policies and protocols, operational components, facilities and resources, communications, transportation, prehospital care, hospital care facilities, and rehabilitation.

## System finance

System support is a complex process that requires government and private funding, community shares, and the judicious use of available resources.^34^ The government is responsible for the provision of quality trauma care to all seriously injured individuals starting from prevention, prehospital care, hospital care, and rehabilitation. Funding can be facilitated further by developing a funding framework by the lead agency and the corporation. Private companies supplying materials and equipment for system operation are also obliged to share in funding the lead agency, educational and training programs, and other activities as necessary. Various government agencies and the private sector players concerned with the care of the injured should develop their own funding plans in coordination with the lead agency and other trauma system leaders.

An operational budget plan for each component in the trauma system should be developed to adequately fund the trauma system's administrative and program priorities. Trauma system expenses must be linked ultimately to a description of the cost-effectiveness of trauma care and its demonstrated benefit to society, such as the numbers of lives saved and of injured persons returned to maximum preinjury productivity. This information will assist decision makers and the public in understanding the relationship between trauma system costs and the system's value to society and will generate support for sustained trauma system funding that may include health insurance, a surcharge of all vehicle registration, licenses, and violation penalties.

Costs of the trauma system are usually estimated through the three phases of care: prehospital, hospital, and rehabilitation.^36^ Prehospital costs can be estimated by calculating the total number of injuries per year, the injury being minor or major, and the average cost per case. Hospital costs also depend on the number and category of the hospitals, such as level I, II, III, or IV. Hospital costs are estimated based on average trauma care costs per individual per year in the emergency department, intensive care unit, intermediate care unit, and regular wards. The average hospital stay for a trauma patient is about 15 days. Added to that are other cost estimations per year, including tests, surgeries, and other ancillary services. Rehabilitation costs can be estimated based on average costs per individual per number of cases per year. Staffing costs may be divided into management, clinical, and training. Management cost estimations include the annual salary for trauma directors, nurse coordinators, and clerical/office expenses. Clinical staffing costs depend on the volume of trauma patients received and the demand per hospital category. Recruitment and training costs are also important to consider in any trauma system.

## Injury prevention

Injury prevention is the most important aspect of trauma system development.^33^ More than half of trauma deaths occur within minutes from injury due to major organ disruptions that are not salvageable even with the best acute trauma care facilities. This portion of the injured population is best addressed by injury prevention.^38^ Preventive efforts should focus on identified local risk factors and high-risk population groups. Strategies that can alter personal behavior through education and legislation have the greatest impact on trauma prevention.^39^ Injury prevention efforts can be categorized into three phases:^40^ primary (precrash phase), e.g., trauma prevention education and law enforcement; secondary (crash phase), e.g., wearing a seat belt; tertiary (postcrash phase), e.g., providing injured victims with optimal prehospital, hospital, and rehabilitative care. These phases involve factors related to human beings themselves, vehicles and equipment, and the environment (Table 1).

**Table 1:** Injury prevention (The Haddon matrix).^38^

Phase		Factors		
		Human	Vehicles and equipment	Environment
Precrash	Crash prevention	Information Attitudes Impairment Police enforcement	Road worthiness Lighting Braking Handling Speed management	Road design and road layout Speed limits Pedestrian facilities

Crash	Injury prevention during the crash	Use of restraints Impairment	Occupant restraints Other safety devices Crash protective design	Crash-protective roadside objects

Postcrash	Life sustaining	First-aid skill Access to medics	Ease of access Fire risk	Rescue facilities Congestion

## Prehospital care

Prehospital care involves prompt communication and activation of the system, proper actions at the scene of the crash by bystanders, and the prompt response of the system. This includes all the appropriate personnel, assessment, and treatment of the injured people at the scene, and transport to trauma care facilities while delivering the necessary medical care before arrival at the hospital. Training is the most important aspect of successful prehospital care. In many communities, prehospital care is provided by “basic first-aid providers” or “Emergency Medical Technicians-Basic” (EMT-Basic). Some centers have intermediate prehospital trauma care providers (EMT-Intermediate) who are able to administer intravenous therapy and apply pneumatic antishock garments in addition to basic maneuvers. A few centers have advanced prehospital training known as EMT-Advanced. Advanced EMTs are able to do endotracheal intubation, needle thoracostomy, and cricothyroidostomy.^41^ The Prehospital Trauma Life Support (PHTLS) program provides basic and advanced prehospital training.^42^ In addition to the importance of prehospital care provision at the scene, prompt and proper transportation to appropriate trauma care facilities are also important for better survival and outcome.^41^ The “golden hour” is an approximate time frame concept emphasizing the urgency necessary for successful management of the injured patient from the time of accident to the receipt of definitive care at the hospital. It is an opportunity window for prehospital and hospital care providers to coordinate medical care with decreased impact from mortality and morbidity due to life-threatening airway, breathing, and circulatory injuries.^43^,^44^ EMT-basic and PHTLS programs are available in Saudi Arabia; however, trained personnel are not up the desired standards for local and national needs.

## Definitive care and rehabilitation

Quality definitive trauma care is the hallmark of trauma systems. Reported improvements in mortality and morbidity from trauma were mostly attributed to designated trauma centers.^32^,^45^ Trauma centers should also participate in the essential activities of a trauma system, including prevention, performance improvement, data submission to trauma registries, representation in the trauma advisory committee, and mutual operational agreement with other regional hospitals to address interfacility transfer, educational support, and outreach.^37^ In most trauma systems, a combination of levels of designated trauma centers will coexist.^46^ A level I trauma center is a sophisticated definitive care facility central to the trauma system, where all severe and complex injuries are managed by an inhouse attending surgeon, other medical and administrative personnel, and ancillary services around the clock. The level I trauma center is a leader of state-of-the-art trauma care that includes quality improvement, education, and research activities. A level II trauma center is also expected to manage severe injuries with the availability of an attending surgeon and other medical personnel; however, graduate education and research are not required. Level III trauma care facilities are usually large community hospitals that can survey, resuscitate, and stabilize all trauma victims even if they need definitive surgery; however, complex cases beyond the available resources are transferred to higher centers with transfer agreements and protocols. Level IV is usually a rural trauma care facility where injured victims can receive initial assessment and management until they become stable enough for safe transfer to higher centers. In an inclusive trauma system, injured patients should be triaged and transported to an appropriate trauma care facility, not necessarily to the nearest health care facility, but to one based on injury severity. Rehabilitation of residual disability from severe injuries is an integral component of the trauma system. The goals of rehabilitative interventions are: to resume normal intellectual and physical functions; to reduce disabilities; and to avoid handicap as far as possible. Ideally, rehabilitation should be planned to start as early as possible from admission time, continue during the acute phase of care, and end if necessary in the chronic rehabilitation center. Rehabilitation representation in the trauma advisory committee is essential for the assessment of needs and national or regional distribution of rehabilitative care, provision of personnel and rehabilitation specialists, resources provision and allocation, interfacility transfer agreements, and research.^37^

## Human resources (education and training)

Human resources investment involves the recruitment and retention of qualified trauma care personnel within all components of the trauma system. Specific educational and training requirements should be delineated through administrative policies and procedures based on national or regional needs of the system. These requirements are usually achieved in collaboration with national or international academic institutions and universities. Varied trauma programs that address almost all aspects of trauma management for physicians, nurses, paramedics, and other personnel are available nationally and internationally.^47^ The periodic review of both the required and supplemental educational opportunities is an activity for the trauma-specific national multidisciplinary, multi-agency advisory committee.^34^ There is a growing concern that patient access to trauma care facilities is becoming more difficult and that interest in trauma training and specialization is declining.^48^ Acute care surgery that deals with all surgical emergencies may help in addressing the shortage of trauma surgeons.^49^

## System evaluation and quality improvement

An effective system evaluation addresses two aspects: patient care and social components.^34^ Patient care includes operational and clinical components in prehospital, hospital, and rehabilitation environments. Social components include legislation, prevention programs, education, research, economics, and an assessment of value of quality in relation to cost. The management information system (MIS) is a comprehensive data resource derived from various components of the trauma system, including trauma registries, prehospital registry, incident after-action reports, injury registry, death certificates, hospital administrative data sets, medical examiner's reports, crash reports, and other data. The MIS is reviewed and studied by the trauma advisory committee and other subcommittees within the system with the aim of driving public policy; enhancing system performance; providing guidance for injury prevention activities and education of trauma care providers; identifying and evaluating system best practices; identifying and evaluating gaps in the system; reassessing trauma resource utilization; tracking patient outcomes; developing performance standards; and measuring system performance against similar systems. The quality of medical care has evolved over the years from simple medical audits, through performance improvement and quality assurance, to quality improvement (QI).^47^ The same thing can be applied to trauma QI, which involves mortality and morbidity meetings, preventable death studies, audit filters, complications, and unexpected (risk adjusted) mortality. For the methods noted above, the primary principle is to identify problems that arise due to correctable factors.^50^,^51^ Corrective actions should be taken to improve these problems and the effects of these actions are evaluated to assess whether they have been successful in correcting the problem. Other methods of trauma QI involve prehospital care reviews, hospital inspection, and continuing education. The QI program is an integral part of the trauma system that contributes to improvement in the trauma care process, decreases mortality, and decreases costs.^10^

## Research

Research is the core of trauma care systems.^34^ It drives the system and provides the foundation for system development and performance improvement.^37^ Research data from all components of the system should be reliable and made available to all investigators. Competitive grants or contracts made available through lead authorities or constituencies should provide funds to support research activities. The trauma registry^52^ provides investigators with information about patient demographics, prevalence and incidence of injuries, other injury details, patterns of care provided within the system, and outcomes. The lead agency is responsible for administration, confidentiality, security, and access to trauma registries. Other population-based data that are not usually entered into trauma registries (such as deaths at the scene, patients managed at nontrauma designated hospitals, or patients with injuries that are not severe enough to require admission) are also important to capture and document for comprehensive system development and research. Multidisciplinary participation in research is essential for the reliability and validity of the research work, which reflects all components of the system, and not only the medical care. Investigators from various disciplines should be selected carefully by the lead agency based on their qualifications and expertise in research in collaboration with academic centers and public health agencies. Analyzed data, conclusions, recommendations, and alterations in the system should be published in peer-reviewed journals and communicated to the public through presentations, annual reviews, and public information formats.

## Conclusion

Trauma in Saudi Arabia is a major public health problem with increasing rates of mortality and morbidity. The socioeconomic burden, depletion of human resources, the emotional and psychological stress on families, and the strain on healthcare facilities are also increasing. To minimize this impact, a national multidisciplinary trauma system has to be developed and implemented before it is too late to manage the further complexities of trauma in the future.
